# Central release of nitric oxide mediates antinociception induced by aerobic exercise

**DOI:** 10.1590/1414-431X20144160

**Published:** 2014-12-19

**Authors:** G.S. Galdino, I.D. Duarte, A.C. Perez

**Affiliations:** 1 Curso de Fisioterapia, Escola de Enfermagem, Universidade Federal de Alfenas, Alfenas, MG, Brasil; 2 Departamento de Farmacologia, Instituto de Ciências Biológicas, Universidade Federal de Minas Gerais, Belo Horizonte, MG, Brasil

**Keywords:** Nitric oxide, Exercise, Pain, Antinociception

## Abstract

Nitric oxide (NO) is a soluble gas that participates in important functions of the central nervous system, such as cognitive function, maintenance of synaptic plasticity for the control of sleep, appetite, body temperature, neurosecretion, and antinociception. Furthermore, during exercise large amounts of NO are released that contribute to maintaining body homeostasis. Besides NO production, physical exercise has been shown to induce antinociception. Thus, the present study aimed to investigate the central involvement of NO in exercise-induced antinociception. In both mechanical and thermal nociceptive tests, central [intrathecal (*it*) and intracerebroventricular (*icv*)] pretreatment with inhibitors of the NO/cGMP/K_ATP_ pathway (L-NOArg, ODQ, and glybenclamide) prevented the antinociceptive effect induced by aerobic exercise (AE). Furthermore, pretreatment (*it*, *icv*) with specific NO synthase inhibitors (L-NIO, aminoguanidine, and L-NPA) also prevented this effect. Supporting the hypothesis of the central involvement of NO in exercise-induced antinociception, nitrite levels in the cerebrospinal fluid increased immediately after AE. Therefore, the present study suggests that, during exercise, the NO released centrally induced antinociception.

## Introduction

Nitric oxide (NO) is a soluble gas continuously synthesized from the amino acid L-arginine in endothelial cells by the constitutive calcium calmodulin-dependent enzyme nitric oxide synthase (NOS) (1). A family of enzymes known as NOS catalyzes this reaction. There are two constitutive forms of the enzyme, neuronal NOS (nNOS) and endothelial NOS (eNOS), and an inducible form, inducible NOS (iNOS) (2). The presence of NOS in the brain was later confirmed (3), this enzyme was subsequently purified (4), and its cDNA was cloned and sequenced (5). Today, it is known that, in the central nervous system (CNS), NO production is associated with cognitive function, its role extending from the induction and maintenance of synaptic plasticity to sleep control, appetite, body temperature, and neurosecretion (6).

In addition to these functions, since the early 1990s studies have demonstrated that NO produces analgesia (7). The first studies showed that the antinociception induced by acetylcholine (ACh) is mediated by the release of NO (7). The antinociceptive effect produced by ACh was blocked by an inhibitor of the formation of NO from L-arginine [N^G^-monomethyl-L-arginine (L-NMMA)] and by methylene blue, an inhibitor of guanylate cyclase (7). Thus, these authors found that an intracellular signaling pathway participates in the analgesia produced by NO.

At the spinal level, NO is concentrated in the dorsal horn of the spinal cord, derived from diverse sources (including glial cells), and it has a definite role in spinal cord circuits (8). Additionally, it has been demonstrated that this neurotransmitter participates at a central level in antinociception via different antinociceptive agents (9).

Lorenzetti and Ferreira (10) found that NO participates in dipyrone-mediated antinociception at the spinal level. These authors have demonstrated that intraplantar administration of L-NMMA abolished the antinociception produced by dipyrone (intraplantar) in rats (10). At the supraspinal level, Tesser-Viscaíno et al. (11), in a model of temporomandibular joint arthritis, demonstrated that NO from nNOS spinal trigeminal neurons plays a role in antinociception. Basal NO concentrations have been shown to reduce the release of γ-aminobutyric acid (GABA) in a Ca^2+^ and Na^+^ dependent manner, while high levels of NO increase the release of GABA, an important neurotransmitter involved in pain control (9). However, high levels of NO are responsible for increasing levels of reactive nitrogen oxide species and reactive oxygen species, which can contribute to an indiscriminate impairment of the structural and functional integrity of cells, and modification of cellular DNA, proteins, and lipids in the brain (12).

Physical exercise is another physiological inductor of NO production. During exercise, the increase in shear stress caused by increasing blood flow and muscle contraction-induced distortion of resistance vessels stimulates eNOS and nNOS (13). Furthermore, microdamage to myofibrils during muscle contractions releases and/or stimulates inflammatory cells, activating iNOS. Red blood cells release ATP in low-oxygen environments and by the deformation caused by muscle contractions. Thus, ATP binds to purinergic receptors on the endothelium, leading to eNOS activation and consequently to NO production (14).

Additionally, recent work by our group demonstrated an involvement of the NO/cGMP/K_ATP_ pathway in peripheral antinociception induced by exercise (15). Exercise-induced analgesia has been demonstrated since the early 1980s (16). However, few central endogenous systems have been described involved in this effect, although recent studies demonstrated the participation of the noradrenergic and endocannabinoid systems (17,18).

Even though several studies have demonstrated the importance of NO in exercise physiology, none have evaluated its central participation. Furthermore, a study would be important that evaluated the involvement of this neurotransmitter in the antinociceptive effect induced by exercise at the central level, whereas NO is released in different areas of the brain and spinal cord (12). Thus, the present study aimed to investigate the central participation of NO in the antinociception induced by aerobic exercise (AE), which can lead to future clinical and experimental studies about mechanisms involved in pain control by non-pharmacological treatments.

## Material and Methods

### Animals

The experiments were performed with male Wistar rats weighing 180-200 g obtained from CEBIO-UFMG-Brazil. The rats were housed in a temperature-controlled room (23±1°C) on an automatic 12:12-h light-dark cycle (6:00 am to 6:00 pm). All tests occurred during the light phase (8:00 am to 4:00 pm). Food and water were freely available until the onset of the experiments. The Ethics Committee on Animal Experimentation of the Universidade Federal de Minas Gerais (protocol #185/2007) approved the study, and all experiments followed the guidelines of the International Association for the Study of Pain on the use of laboratory animals.

### Drugs

The drugs used were *N*-nitro-L-arginine (L-NOArg; Sigma, USA), an unspecific NOS inhibitor; aminoguanidine (AMG, Sigma), an iNOS inhibitor; *N* ^5^-(1-iminoethyl)-L-ornithine dihydrochloride (L-NIO; Sigma), an eNOS inhibitor; and *N*ω-propyl-L-arginine (L-NPA; Cayman, USA), an nNOS inhibitor, all diluted in physiological saline solution (0.9% NaCl); and 1H-(1,2,4)oxidiazolo[4,3-a]quinoxalin-1-one (ODQ; Tocris, USA), a guanylyl cyclase inhibitor, diluted in DMSO (10% in saline solution); and glibenclamide (GLB; Sigma), a K_ATP_ channel blocker, diluted in Tween (1% in saline solution). The control group received the same volume of physiological saline or vehicle in the same area as the experimental groups. The concentrations of drugs were selected according to a dose-response curve (data not shown).

### Injections

#### Intrathecal injection

The intrathecal injections (*it*) were performed in a volume of 10 µL in the subarachnoid space between L5 and L6 using a 30 G×1/2-inch needle and a 50-µL precision syringe (Hamilton Company, USA) (19). Intrathecal injection is stressful for rats and, according to the International Association for the Study of Pain (IASP) recommendations, requires anesthesia. Thus, before injection, rats were slightly anesthetized with volatile isoflurane (3.5%) and recovered 5 min after removal from the anesthesia chamber. Correct *it* positioning of the needle tip was confirmed by a characteristic tail-flick response in the animal. Lidocaine (4%, 10 µL) was administered to a group of test animals, using temporary paralysis of the hind limbs as an endpoint to confirm the effectiveness of the injection technique. Intrathecal injections were administered immediately prior to exercise.

#### Intracerebroventricular injection

Initially before intracerebroventricular (*icv*) injections (20), each rat was anesthetized with a mixture of ketamine (80 mg/kg) and xylazine (10 mg/kg) injected *ip*, and then placed in a stereotaxic apparatus (Stoelting, USA). The scalp was incised, and the skull was leveled off around the bregma. A 22-gauge, 12-mm stainless-steel guide cannula was inserted into the right lateral ventricle of the brain. The cannula aimed for the following coordinates: 1.5 mm posterior to the bregma, 2.5 mm lateral to the midline, and 3.3 mm below the top of the skull (21). The skull was fixed to the cannula using three screws and acrylic dental floss. A 12.5-mm stylet was then inserted into the cannula to keep it patent before the injection. Animals had a 5-day recovery period before the experiments. For *icv* drug injections, a 12.5-mm injection needle attached to a 30-cm polyethylene tube fitted to a 10-µL Hamilton syringe was used. Then, the stylet was withdrawn manually, and the injection needle was manually inserted into the guide cannula. The volume of solution injected into the lateral ventricle was 5 µL over a period of 120 s. Intracerebroventricular injections were performed immediately prior to exercise.

### Exercise

Acute AE was performed using a rodent treadmill. Animals ran with a progressive speed of 20 m/min and 0% inclination, for an average time of 45.03±2 min, until fatigue (15). Fatigue was defined as the point at which the animals were unable to keep pace with the treadmill. The back of the treadmill had an electrical stimulator (3 V) to encourage the animals to run. To familiarize the rats to exercise, thereby reducing the effects of stress, they were run daily on the treadmill.

The groups were as follows (N=6 per group): control (Co), animals that did not perform exercise and received saline; acute AE (AE), animals that ran and received saline; AE+L-NOArg, animals pretreated with unspecific NOS inhibitor that exercised; AE+ODQ, animals pretreated with guanylyl cyclase inhibitor that exercised; AE+GLB, animals pretreated with K_ATP_ channel blocker irreversible (glibenclamide) that exercised; AE+AMG, animals pretreated with iNOS inhibitor (aminoguanidine) that exercised; AE+L-NIO, animals pretreated with eNOS inhibitor; and AE+L-NPA, animals pretreated with nNOS inhibitor. Different groups of animals received the drugs via *it* and *icv* administration. In each route of administration (*it* or *icv*), the control group was the same in the experiments performed with unspecific or specific inhibitors of the NO/cGMP/K_ATP_ pathway. One group received the same quantity of electrical stimulation as the AE group, and there was no change to the nociceptive threshold.

### Paw-withdrawal test

An apparatus (Ugo Basile, Italy) was used to evaluate a response to mechanical nociceptive stimuli at central and peripheral levels. A cone-shaped pusher with a rounded tip (base diameter=9 mm) was applied to the plantar surface of the animal's paw. The frequency of force application was set at 150-160 g/s, and there was a 240 g/s loading cutoff to avoid damaging the tissue. The intensity of pressure causing an escape reaction was defined as the withdrawal threshold (22).

### Tail-flick test

Animals were placed on the tail-flick apparatus (Ugo Basile), which allows collection of information on the mechanism and location of the antinociceptive activity detected, since the tail-flick reflex is spinally integrated (23). In this apparatus, the animal's tail is smoothed into a groove that contains a photocell. A light source was activated and the light remained focused on the tail until the rat moved its tail (a spinal reflex), thereby switching the light off. The intensity of the light was adjusted to obtain a baseline tail-flick latency of 2-4 s, and a cutoff time of 9 s was chosen to prevent tissue damage (23).

### Cerebrospinal fluid (CSF) collection

CSF was collected while the rats were under anesthesia with 2% isoflurane by puncture between the occipital protuberance and the spinal atlas into the cisterna magna with a 30-gauge needle (13×3 in) (24). The material was centrifuged for 5 min (2839.2 *g*) and stored in a freezer at -80°C. In the exercised group (AE), CSF and plasma were collected immediately after exercise.

### Nitrite determination

Nitrite levels were measured using the Griess reaction (25). Briefly, 100 µL of the homogenate was applied to a microliter plate well, followed by 100 µL of Griess reagent [0.2% (w/v) naphthylene ethylenediamine and 2% (w/v) sulfanilamide in 5% (v/v) phosphoric acid]. After 10 min of color development at room temperature, the absorbance was measured with a microplate reader (Titertek Multiskan MCC/340; Flow Laboratories, USA) at a wavelength of 545 nm. Each sample was assayed in duplicate wells. The nitrite standard reference curves were made with sodium nitrite in distilled water at concentrations of 100, 50, 25, 12.5, 6.25, 3.13, and 1.56 μM. The detection limit of the assay was ∼1.5 μmol/L in distilled water.

### Statistical analysis

Data are reported as means±SE of the evaluated parameter and were analyzed for statistical significance by one-way ANOVA followed by the Bonferroni *post hoc* test for multiple comparisons. Comparisons between two groups (*t*-test) were used for results obtained by nitrite determination. The minimum level of significance was considered to be P<0.05. Statistical analyses and preparation of figures were performed using the GraphPad Prism software, version 4 (USA).

## Results

**Figure 1:**
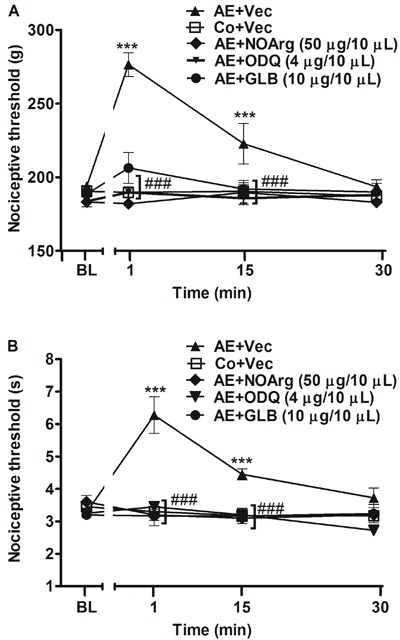
Effect of intrathecal administration of nitric oxide/cGMP/K_ATP_ pathway inhibitors on the antinociception induced by acute aerobic exercise (AE) in the paw-withdrawal (*A*) and tail-flick (*B*) tests. Rats were pretreated with intrathecal injection of *N*-nitro-L-arginine (L-NOArg, 50 μg/10 µL), 1H-(1,2,4)oxidiazolo[4,3-a]quinoxalin-1-one (ODQ, 4 μg/10 µL), and glibenclamide (GLB, 10 μg/10 µL) immediately before the onset of AE, which lasted for a mean of 45.3±2.0 min. Mechanical and thermal nociceptive thresholds were measured before and after 1, 15, 30 min of AE. Data are reported as means±SE of 6 animals per group. ***P<0.001, compared to the control group (Co); ^###^P<0.001, compared to the AE group (one-way ANOVA followed by the Bonferroni test). Vec: vehicle; BL: baseline latency.

**Figure 2:**
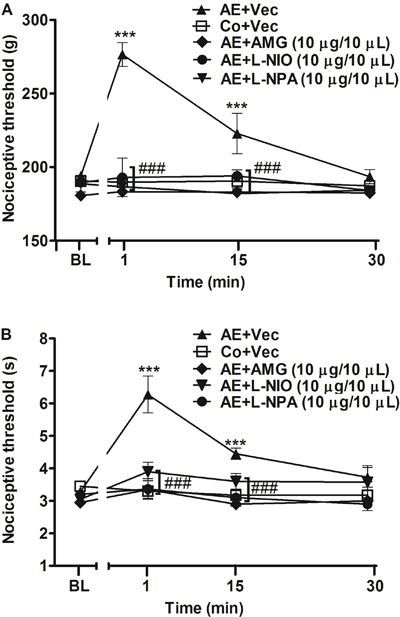
Effect of intrathecal administration of specific NOS inhibitors on the antinociception induced by acute aerobic exercise (AE) in the paw-withdrawal (*A*) and tail-flick (*B*) tests. Rats were pretreated with intrathecal injection of aminoguanidine (AMG, 10 μg/10 µL), *N* ^5^-(1-iminoethyl)-L-ornithine dihydrochloride (L-NIO, 10 μg/10 µL) and *N*ω-propyl-L-arginine (L-NPA, 10 μg/10 µL) immediately before the onset of exercise, which lasted for a mean of 42.2±4.0 min. Mechanical and thermal nociceptive thresholds were measured before and after 1, 15, 30 min of AE. Data are reported as means±SE of 6 animals per group. ***P<0.001, compared to the control group (Co); ^###^P<0.001, compared to the AE group (one-way ANOVA followed by the Bonferroni test). Vec: vehicle; BL: baseline latency.

**Figure 3:**
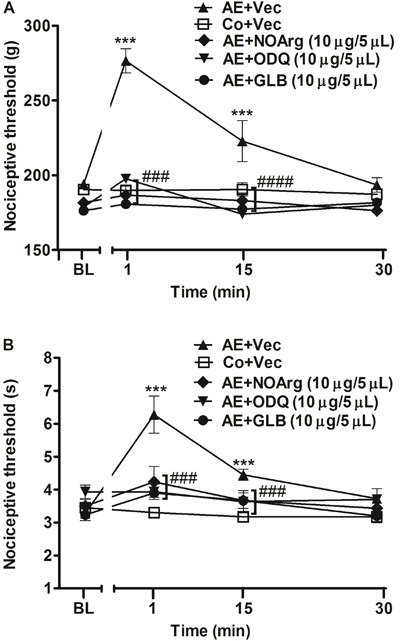
Effect of intracerebroventricular administration of nitric oxide/cGMP/K_ATP_ pathway inhibitors on the antinociception induced by acute aerobic exercise (AE) in the paw-withdrawal (*A*) and tail-flick (*B*) tests. Rats were pretreated with intracerebroventricular injection of *N*-nitro-L-arginine (L-NOArg, 10 μg/5 µL), H-(1,2,4)oxidiazolo[4,3-a]quinoxalin-1-one (ODQ, 10 μg/5 µL) and glibenclamide (GLB, 10 μg/5 µL) immediately before the onset of exercise, which lasted for a mean of 44.2±1.5 min. Mechanical and thermal nociceptive thresholds were measured before and after 1, 15, 30 min of AE. Data are reported as means±SE of 6 animals per group. ***P<0.001, compared to the control group (Co); ^###^P<0.001, compared to the AE group (one-way ANOVA followed by the Bonferroni test). Vec: vehicle; BL: baseline latency.

Immediately after AE, the nociceptive threshold of rats was increased (P<0.05) for more than 15 min in both paw-withdrawal and tail-flick nociceptive tests (Figures 1, 2, and 3). The increase was prevented (P<0.001) by inhibitors of the NO/cGMP/K_ATP_ pathway, the unspecific NOS inhibitors, L-NOArg (50 µg/10 µL), ODQ (4 µg/10 µL), and GLB (10 µg/10 µL), preinjected *it* (Figure 1A and B). Furthermore, preinjection *it* of specific NOS inhibitors, L-NIO, AMG, and L-NPA, also significantly (P<0.001) prevented exercise-induced antinociception in both paw-withdrawal and tail-flick tests (Figure 2A and B).

**Figure 4:**
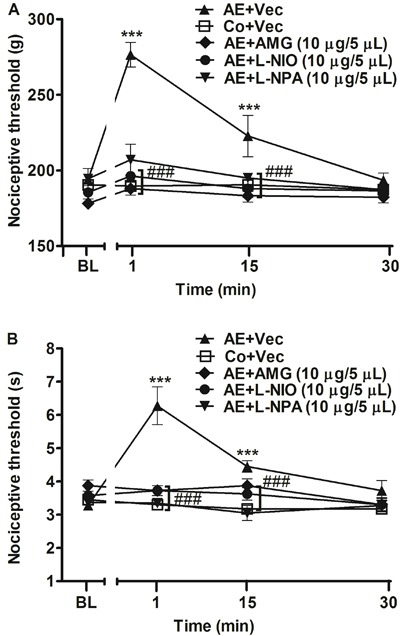
Effect of intracerebroventricular administration of specific NOS inhibitors on the antinociception induced by acute aerobic exercise (AE) in the paw-withdrawal (*A*) and tail-flick (*B*) tests. Rats were pretreated with intracerebroventricular injection of aminoguanidine (AMG, 10 μg/5 µL), *N* ^5^-(1-iminoethyl)-L-ornithine dihydrochloride (L-NIO, 10 μg/5 µL) and *N*ω-propyl-L-arginine (L-NPA, 10 μg/5 µL) immediately before the onset of exercise, which lasted for a mean of 43.3±1.0 min. Mechanical and thermal nociceptive thresholds were measured before and after 1, 15, 30 min of AE. Data are reported as means±SE of 6 animals per group. ***P<0.001, compared to the control group (Co); ^###^P<0.001, compared to the AE group (one-way ANOVA followed by the Bonferroni test). Vec: vehicle; BL: baseline latency.

Additionally, to confirm the supraspinal involvement of the NO/cGMP/K_ATP_ pathway, the same inhibitors were injected *icv*. Figure 3A and B shows that preinjection of NO/cGMP/K_ATP_ pathway inhibitors L-NOArg (10 µg/5 µL), ODQ (10 µg/5 µL), and GLB (10 µg/5 µL) prevented (P<0.001) the antinociceptive effect produced by AE in the paw-withdrawal and tail-flick tests. Similar to results found with the drugs administered *it*, the specific NOS inhibitors L-NIO, AMG, and L-NPA also prevented (P<0.001) exercise-induced antinociception in both nociceptive tests (Figure 4A and B).

**Figure 5:**
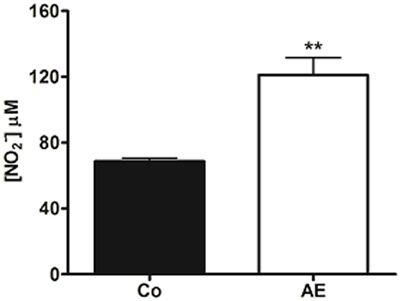
Effect of acute aerobic exercise (AE) on nitrite [NO_2_] cerebrospinal fluid (CSF) levels. Immediately after AE (open bar), the cerebrospinal fluid levels were increased compared to the control group (Co, shaded bar). Data are reported as means±SE of 5 animals per group. **P<0.01, compared to Co (non-exercised rats) (*t*-test).

Furthermore, the nitrite levels in CSF were significantly (P<0.01) increased immediately after AE (Figure 5), supporting the hypothesis that the NO released centrally during exercise takes part in the antinociception.

## Discussion

The present study found that the NO/cGMP/K_ATP_ pathway possibly participates in exercise-induced antinociception at both spinal and supraspinal levels. Studies have demonstrated the involvement of endogenous mechanisms involved in this effect (16). However, most of the studies evaluated the peripheral influence of these mechanisms in exercise-induced analgesia. Only two studies demonstrated the participation of endogenous substances in this effect at the central level (17,18). These studies reported a reversion of the antinociceptive effect produced by exercise after *it* and *icv* administration of noradrenergic and cannabinoid receptor antagonists. Furthermore, those authors demonstrated that, after exercise, there was an increase in noradrenergic and cannabinoid receptor expression.

According to our previous studies and evidence in the literature that demonstrated a correlation of both systems (noradrenergic and endocannabinoid) with NO, our group aimed to investigate the central involvement of the NO/cGMP/K_ATP_ pathway in this effect. In support of this, Romero et al. (26) showed that the antinociception produced by endocannabinoid *N*-palmitoyl-ethanolamine was antagonized by specific inhibitors of the NO/cGMP pathway. In addition to this, NO is known to react with norepinephrine *in vivo* in the brain to form 6-nitro-norepinephrine, which inhibits neuronal norepinephrine reuptake. A study corroborating this found that *it* injection of 6-nitro-norepinephrine produced antinociception and interacted additively with norepinephrine in this effect, suggesting a functional interaction between spinal NO and norepinephrine in analgesia (27). Furthermore, it has been reported that NO also increases the release of norepinephrine in various brain areas (28). Although it was not the aim of our study, NO may be activated by both systems previously described, during exercise.

The results presented in this study demonstrated that the three forms of NOS (nNOS, eNOS, and iNOS) participated in the antinociceptive mechanism. When preadministered *it*, the antagonists of the three forms of NOS reversed the antinociceptive effect produced by exercise, supporting our hypothesis involving both forms in this effect. Other research has shown that NO donors inhibited spontaneously activated neurons in the superficial dorsal horn of rats (29) and diminished evoked substance P and calcitonin gene-related peptide released in spinal cord slices *in vitro*, effects that are consistent with analgesic actions (30). In addition, NOS has been identified in glial cells, interneurons, and fibers in the spinal cord (31). In support of this, several factors are responsible for the increase in NO production during resistance exercise. In rodent skeletal muscle, nNOS, eNOS, and iNOS isoforms are highly expressed within muscle fibers and activated by exercise. The increase in shear stress caused by increased blood flow and muscle contraction-induced distortion of resistance vessels stimulates eNOS and nNOS (13). In addition, microdamage in the myofibrils during muscle contractions releases and stimulates inflammatory cells that will activate iNOS present in red blood cells, which will release ATP in low-oxygen environments and in response to deformation of muscle during contractions. Then, ATP binds to purinergic receptors on the endothelium, leading to eNOS activation and NO production (14). Furthermore, nNOS and eNOS transcription and expression were found to be increased in human skeletal muscle after exercise (32). Additionally, it was reported that exercise induces nNOS, eNOS, and iNOS expression in the CNS (33). Thus, the NO release during exercise may happen systemically, inclusive at the central level.

Our results also showed that the three NO isoforms participated in exercise-induced antinociception at the supraspinal level, after a reversion of the antinociceptive effect by preadministration *icv* of specific inhibitors. In addition, studies have demonstrated that NO has a complex and diverse role in the modulation of nociceptive processing at various levels of the neuraxis (34). A study reported that swimming for 2 h/day produced an increase in iNOS, eNOS, and nNOS expression in the hippocampus (35). NO has also been found in neurons in the periaqueductal grey matter (PAG), an important area of pain modulation. In addition, the dorsolateral and ventrolateral PAG contains a column of NOS-containing cells, which may release NO that could participate in the inhibitory modulation of pain (36). NO might also promote the release of serotonin, an important neurotransmitter involved in the inhibition of nociceptive impulses in the dorsal horn of the spinal cord (37). In accordance with the above, we suggest that the central antinociceptive effect produced by exercise occurred by activation of descending control of pain associated to NO activation and production. In addition, to support our results, an increase in nitrite levels in the CSF was found. Thus, we suggest that both NOS isoforms can be activated at the same intensity by the exercise protocol used.

NO may stimulate guanylyl cyclase-coupled NO receptors in axons, leading to increasing cGMP levels in axons of the CNS (9). Our results showed that pretreatment with a cGMP inhibitor (ODQ) prevented the antinociception induced by exercise.

K_ATP_ channels play an important role in supraspinal, spinal, and peripheral antinociception. The opening of these channels for openers (monoxidil, metamizol, and opioids agonists) elucidated antinociception (38). In addition, K_ATP_ channels are on the surface membranes and mitochondria of many different cell types involved in exercise, including pancreatic β-cells, neurons, cardiac myocytes, skeletal, and smooth muscle cells (38). Furthermore, the K_ATP_ channel blocker glibenclamide reversed the antinociceptive effect of exercise. Similar to our results, morphine-induced antinociception in nondiabetic mice was antagonized by pretreatment with glibenclamide. Thus, we suggest that exercise may induce the expression of these channels in CNS areas that participate in pain modulation.

In conclusion, the results of this study indicated that the NO/cGMP/K_ATP_ pathway participates in exercise-induced antinociception at both spinal and supraspinal levels. Furthermore, it demonstrated that this effect involves the three isoforms of NOS. Thus, the present work is important to further studies on the endogenous mechanisms involved in the antinociceptive effect produced by exercise. Future studies will help unravel possible endogenous mechanisms involving exercise-induced analgesia, which may aid in the clinical treatment of patients with different painful conditions.
